# Polymer-free versus durable polymer drug-eluting stents in patients with coronary artery disease: A meta-analysis

**DOI:** 10.1016/j.amsu.2018.12.003

**Published:** 2018-12-11

**Authors:** James J. Wu, Joshua A.H. Way, Leonard Kritharides, David Brieger

**Affiliations:** a Sydney Medical School, The University of Sydney, Camperdown, Australia; b Department of Cardiology, Concord Repatriation General Hospital, Concord, Australia

**Keywords:** Coronary artery disease, Drug-eluting stents, Durable polymer, Meta-analysis, Polymer-free

## Abstract

**Background:**

Polymer-free drug-eluting stents (PF-DES) were introduced with the aim of reducing the risk of stent thrombosis associated with durable polymer drug-eluting stents (DP-DES). The comparison of safety and efficacy profiles between these two stent platforms remains unclear.

**Materials and methods:**

We conducted electronic database searches for randomized controlled trials (RCTs) comparing patients treated with either PF-DES or DP-DES. Outcomes included definite or probable stent thrombosis (ST), myocardial infarction (MI), cardiac death, all-cause death, target lesion revascularization (TLR), and target vessel revascularization (TVR). A random-effects model was used to derive risk ratios (RRs) with 95% confidence intervals (CIs). Subgroup analyses based on different variables were also performed. After screening a total of 1026 articles, the present meta-analysis included 13 RCTs comprising 8021 patients.

**Results:**

No significant differences were found for the risks of definite or probable ST (RR, 0.94; 95% CI, 0.62–1.43; *P* = 0.77), MI (RR, 1.06; 95% CI, 0.85–1.33; *P* = 0.61), cardiac death (RR, 0.98; 95% CI, 0.80–1.21; *P* = 0.88), all-cause death (RR, 0.87; 95% CI, 0.76–1.00; *P* = 0.06), TLR (RR, 1.12; 95% CI, 0.94–1.33; *P* = 0.22), and TVR (RR, 1.18; 95% CI, 0.87–1.61; *P* = 0.29). Similarly, no significant differences were found for all outcomes regardless of anti-proliferative drug, except for an increased risk of TLR for polymer-free paclitaxel-eluting stents compared with DP-DES (RR, 2.32, 95% CI, 1.30–4.14; *P* = 0.005).

**Conclusions:**

Our findings showed that PF-DES and DP-DES confer equivalent safety and efficacy profiles, with similar rates of stent thrombosis.

## Highlights

• Polymer-free and durable polymer drug-eluting stents confer similar safety and efficacy profiles.
• There were comparable rates of stent thrombosis between the two stent platforms.
• Further trials with greater length of follow-up are warranted to assess long-term safety and efficacy outcomes.

## Abbreviations

BMS: bare metal stents
BP-DES: biodegradable polymer drug-eluting stents
DAPT: dual antiplatelet therapy
DP-DES: durable polymer drug-eluting stents
M-H: Mantel-Haenszel
MI: myocardial infarction
OR: odds ratio
PCI: percutaneous coronary intervention
PF-DES: polymer-free drug-eluting stents
PF-PES: polymer-free paclitaxel-eluting stents
RCT: randomized controlled trial
RR: risk ratio
ST: stent thrombosis
TLR: target lesion revascularization
TVR: target vessel revascularization

## 1 Introduction

Drug-eluting stents have been a major advance in percutaneous coronary intervention (PCI). New developments in coronary stent technology have contributed to improved outcomes of patients with coronary artery disease. The sequential generations of devices have represented significant milestones in stent design, structure, and component materials [1]. These stent platforms have included bare metal stents (BMS), durable polymer drug-eluting stents (DP-DES) and polymer-free drug-eluting stents (PF-DES). First-generation DP-DES were developed to reduce the risk of in-stent restenosis and target lesion revascularization associated with BMS [2]. Despite these promising results, first-generation DP-DES were shown to have an increased risk of very late (>12 months) stent thrombosis compared with BMS [3]. The pathophysiology of stent thrombosis has been attributed to various factors, such as polymer-induced hypersensitivity reaction, stent malapposition, incomplete strut re-endothelialization, and accelerated neoatherosclerosis [4]. To address this issue, a new generation of DP-DES were developed, with improvements in anti-proliferative drugs, polymer coatings, and strut thickness [5]. The introduction of second-generation DP-DES reduced the risk of very late stent thrombosis associated with the preceding generation of devices [6]. Nevertheless, the potential thrombogenic nature of the polymer coating in second-generation DP-DES remains a concern, and suggestions have been made to extend the duration of dual antiplatelet therapy (DAPT) in patients receiving DP-DES [7].

PF-DES were designed to achieve similar advantages of BMS (reduced risk of stent thrombosis) and DP-DES (reduced risk of target lesion revascularization). These devices consist of a microporous metallic stent platform and an inorganic coating that can be loaded with an anti-proliferative drug [8]. The theoretical benefit of PF-DES is the elimination of the need for a polymer coating, which acts as a potential chronic inflammatory stimulus [9]. However, the main challenge for PF-DES has been the attainment of a sufficient level of anti-proliferative drug in the inorganic coating to ensure the inhibition of neointimal hyperplasia and in-stent restenosis [10]. Therefore, we performed a meta-analysis of randomized controlled trials (RCTs) to evaluate the safety and efficacy profiles of PF-DES compared with DP-DES.

## 2 Methods

### 2.1 Selection criteria

In the present meta-analysis, we included RCTs comparing patients with coronary artery disease who were randomized to receive PCI with either PF-DES or DP-DES. We included studies that assessed at least one of the following clinical outcomes: definite or probable stent thrombosis (ST), myocardial infarction (MI), cardiac death, all-cause death, target lesion revascularization (TLR), and target vessel revascularization (TVR). Only the most recent report with the greatest length of follow-up was included when institutions published progressive reports of an ongoing study. Studies that evaluated other types of stents, such as BMS, biodegradable polymer drug-eluting stents (BP-DES), or bioresorbable vascular scaffolds were excluded. Furthermore, studies that only assessed angiographic outcomes were excluded. Electronic database searches were limited to studies that involved human subjects. Conference abstracts, editorials, case reports, and review articles were excluded.

### 2.2 Search strategy

The Preferred Reporting Items for Systematic Reviews and Meta-Analyses (PRISMA) guidelines were used to perform the present meta-analysis [11]. Ovid Medline, PubMed, Cochrane Central Register of Controlled Trials (CCTR), Cochrane Database of Systematic Reviews (CDSR), American College of Physicians (ACP) Journal Club, and Database of Abstracts of Reviews of Effectiveness (DARE) were searched from their dates of inception to October 2018. We identified potentially relevant studies in the electronic database searches using the following keywords or MeSH terms: “randomized controlled trial”, “drug-eluting stent”, “polymer-free”, “durable polymer”, “permanent polymer”, “sirolimus-eluting stent”, “paclitaxel-eluting stent”, “everolimus-eluting stent”, “zotarolimus-eluting stent”, and “stent thrombosis”. Reference lists of retrieved articles were screened and assessed according to the inclusion and exclusion criteria.

### 2.3 Data extraction

We extracted data from texts, tables, figures, and supplementary materials. There were two investigators (JJW and JAW), who independently screened each retrieved article to determine the suitability for inclusion at the title or abstract level. Studies that met the inclusion and exclusion criteria were included for quantitative assessment. Discussion and consensus with the senior author (DB) occurred when there were discrepancies between the two reviewers. We extracted data for the following baseline characteristics: age, sex, diabetes mellitus, hypertension, hyperlipidemia, current smoking, previous MI, previous procedure (PCI and coronary artery bypass grafting), clinical presentation (stable and unstable angina), and target vessel location (left anterior descending, left circumflex, and right coronary artery). The main outcome of interest was definite or probable ST, as defined by the Academic Research Consortium (ARC) [12]. We also extracted data for other clinical outcomes, including MI, cardiac death, all-cause death, TLR, and TVR. All outcomes were assessed at the longest follow-up available.

### 2.4 Critical appraisal and statistical analysis

The included studies were qualitatively assessed using the risk of bias tool, which was proposed by the Cochrane Collaboration [13]. In addition, the risk of publication bias was evaluated by visually estimating funnel plots [14]. Summary statistics and risk estimates were expressed as risk ratios (RRs) with 95% confidence intervals (CIs) for the comparison of patients receiving PF-DES versus DP-DES. Heterogeneity between studies was evaluated using the χ^2^ test. The percentage of total variation across studies was estimated using the I^2^ statistic, with values greater than 50% indicating significant heterogeneity [15]. We used the Mantel-Haenszel (M-H) random-effects model because there were assumed variations in treatment effect between studies. Late safety and efficacy outcomes were assessed using a landmark analysis beyond 1 year of follow-up. We also performed subgroup analyses based on the following variables: PF-DES anti-proliferative drug (amphilimus, biolimus, paclitaxel, sirolimus, or sirolimus/probucol); DP-DES anti-proliferative drug (everolimus, paclitaxel, sirolimus, or zotarolimus); generation of DP-DES (first-generation or second-generation); and duration of DAPT (6 months or 12 months). Statistical analysis was conducted using RevMan Version 5.3 (The Nordic Cochrane Center, The Cochrane Collaboration, Copenhagen).

## 3 Results

### 3.1 Study selection

**Fig. 1:**
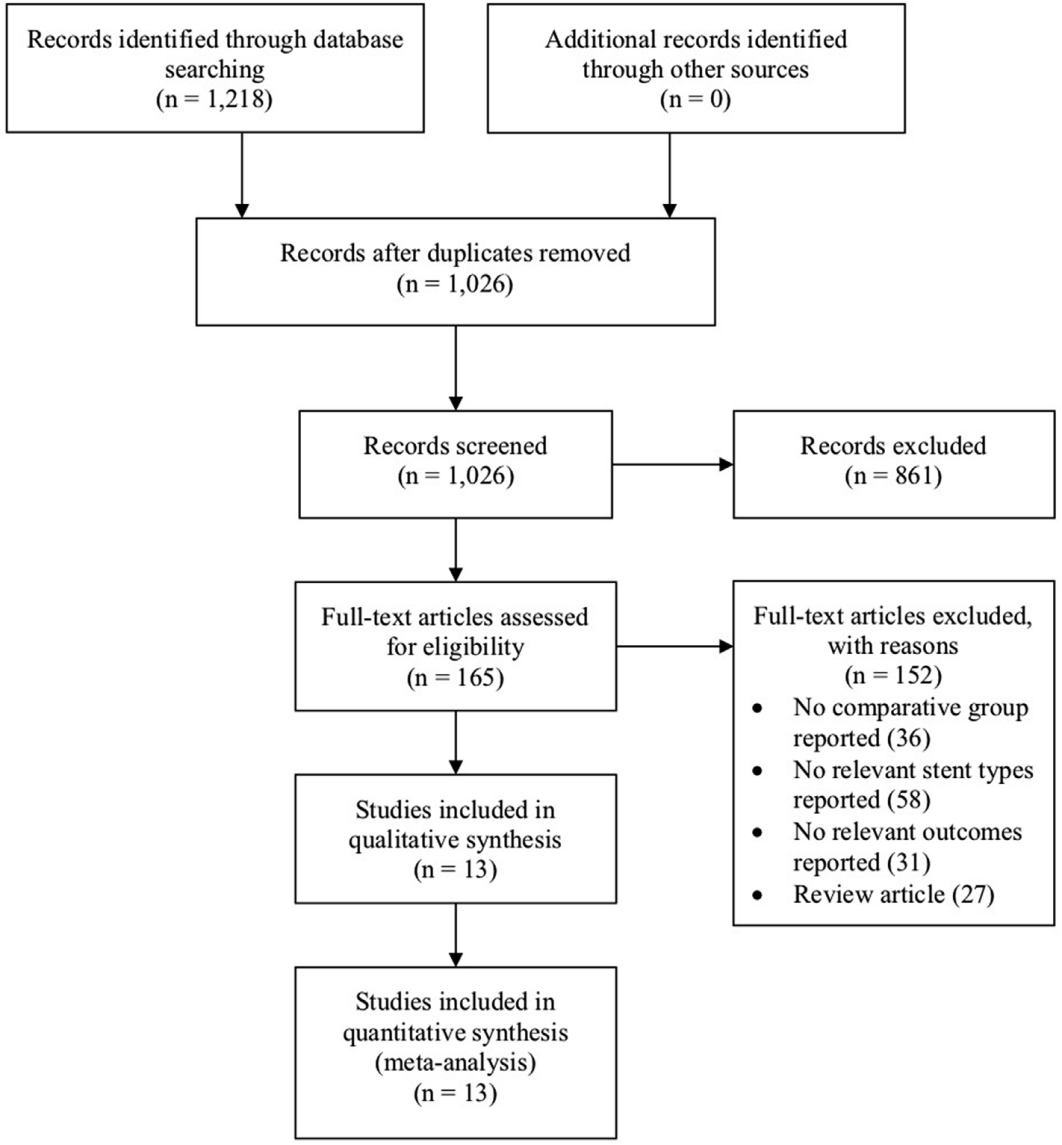
Study selection process.

Fig. 1 shows the study selection process. A total of 1218 references were identified through electronic database searches. After duplicate references were removed, we retrieved 1026 potentially relevant articles. In the present meta-analysis, we included 13 RCTs [[16], [17], [18], [19], [20], [21], [22], [23], [24], [25], [26], [27], [28]] consisting of 8021 patients with coronary artery disease who were randomized to receive PCI with either PF-DES (n = 4545) or DP-DES (n = 3476). Table 1 outlines the study characteristics of the included trials. Patients receiving PF-DES were treated with either amphilimus-, biolimus-, paclitaxel-, sirolimus-, or sirolimus/probucol-eluting stent. Patients receiving DP-DES were treated with either everolimus-, paclitaxel-, sirolimus-, or zotarolimus-eluting stent.

**Table 1:** Study characteristics.

Study	Year	Patient (n)		DAPT (months)	Follow-Up (months)	Anti-Proliferative Drug		Outcomes
		PF-DES	DP-DES			PF-DES	DP-DES	
BioFreedom FIM [19]	2016	122	60	6	60	Biolimus	Paclitaxel	Definite or probable ST, MI, cardiac death, all-cause death, TLR, TVR
Dang [20]	2012	50	55	6	12	Paclitaxel	Sirolimus	Definite or probable ST, MI, cardiac death, all-cause death, TLR
ISAR-TEST [21]	2013	225	225	6	60	Sirolimus	Paclitaxel	Definite or probable ST, MI, cardiac death, all-cause death, TLR
ISAR-TEST-2 [16]	2010	333	335	12	24	Sirolimus/Probucol	Sirolimus	Definite or probable ST, MI, all-cause death
ISAR-TEST-3 [17]	2009	201	202	12	24	Sirolimus	Sirolimus	Definite or probable ST, MI, all-cause death, TLR
ISAR-TEST-5 [22]	2016	2002	1000	6	60	Sirolimus/Probucol	Zotarolimus	Definite or probable ST, MI, cardiac death, all-cause death, TLR, TVR
LIPSIA Yukon [26]	2014	118	114	12	60	Sirolimus	Paclitaxel	Definite or probable ST, MI, cardiac death, all-cause death, TLR, TVR
Nano [27]	2014	132	136	12	24	Sirolimus	Sirolimus	Definite or probable ST, MI, cardiac death, all-cause death, TVR
NEXT [18]	2012	148	148	6	12	Amphilimus	Paclitaxel	Definite or probable ST, MI, cardiac death, all-cause death, TLR, TVR
ReCre8 [24]	2018	747	744	12	12	Amphilimus	Zotarolimus	Definite or probable ST, MI, cardiac death, all-cause death, TLR
RESERVOIR [23]	2016	56	56	12	12	Amphilimus	Everolimus	MI, cardiac death
Shiratori [25]	2014	84	80	6	24	Paclitaxel	Paclitaxel	Definite or probable ST, MI, cardiac death, all-cause death, TLR, TVR
Zhang [28]	2013	327	321	12	24	Paclitaxel	Sirolimus	Definite or probable ST, MI, cardiac death, all-cause death, TVR

DAPT = dual antiplatelet therapy; DP-DES = durable polymer drug-eluting stents; MI = myocardial infarction; PF-DES = polymer-free drug-eluting stents; ST = stent thrombosis; TLR = target lesion revascularization; TVR = target vessel revascularization.

The Cochrane Collaboration risk of bias tool assessed the RCTs to be of high quality and appropriate for inclusion in the present meta-analysis, with minimal risk of bias (Supplementary Table 1) [13]. All trials had a randomized and multicenter design that defined the patient populations and clinical outcomes, with a median follow-up of 24 months. One trial [19] included three comparison arms of patients randomized to receive either standard-dose polymer-free biolimus-eluting stents (PF-BES), low-dose PF-BES, or DP-DES. Data for standard- and low-dose PF-BES were pooled together. Two trials [17,28] included a third comparison arm of patients randomized to receive BP-DES. Data from these third comparison arms were excluded, since BP-DES were irrelevant to our research question. Earlier reports of two trials [22,26] were excluded because the institutions published a more recent report with greater length of follow-up.

### 3.2 Patient and procedural characteristics

Table 2 outlines the baseline characteristics of the included trials. The weighted mean age of enrolled patients receiving PF-DES was 66.3 ± 10.9 years and those receiving DP-DES was 66.3 ± 10.6 years. Overall, the two comparison arms had similar proportions of male patients and with comorbidities, previous procedures, clinical presentations, and target vessel locations (all *P* > 0.05).

**Table 2:** Baseline characteristics.

Baseline Characteristic	PF-DES	DP-DES	RR or WMD (95% CI)	*P* Value
Age (years)	66.3 ± 10.9	66.3 ± 10.6	−0.39 (−1.03 to 0.25)	0.23
Male	3403/4572 (74.4)	2617/3503 (74.7)	0.99 (0.97–1.02)	0.49
Diabetes mellitus	1361/4572 (29.8)	1025/3503 (29.3)	1.00 (0.97–1.03)	0.80
Hypertension	3002/4572 (65.7)	2258/3503 (64.5)	1.01 (0.98–1.04)	0.49
Hyperlipidemia	2545/4452 (57.2)	1939/3387 (57.2)	0.97 (0.93–1.00)	0.08
Current smoking	1028/4432 (23.2)	794/3367 (23.6)	1.06 (0.98–1.15)	0.15
Previous MI	1120/4572 (24.5)	846/3503 (24.2)	0.97 (0.89–1.04)	0.38
Previous PCI	338/1807 (18.7)	353/1745 (20.2)	0.90 (0.79–1.02)	0.10
Previous CABG	352/4095 (8.6)	258/3079 (8.4)	1.00 (0.85–1.17)	0.98
Clinical presentation				
Stable angina	1691/3254 (52.0)	1001/2181 (45.9)	1.04 (0.97–1.11)	0.26
Unstable angina	1125/3767 (29.9)	861/2700 (31.9)	0.95 (0.89–1.01)	0.09
Target vessel location				
Left anterior descending	2444/5177 (47.2)	1809/3672 (31.9)	0.96 (0.91–1.02)	0.24
Left circumflex	1373/5177 (26.5)	983/3672 (26.8)	1.04 (0.94–1.14)	0.43
Right coronary	1691/5177 (32.7)	1195/3672 (32.5)	1.03 (0.97–1.09)	0.35

Values are n/N (%) or mean ± SD; DP-DES = durable polymer drug-eluting stents; PF-DES = polymer-free drug-eluting stents; RR, risk ratio; WMD = weighted mean difference.

### 3.3 Definite or probable stent thrombosis

**Fig. 2:**
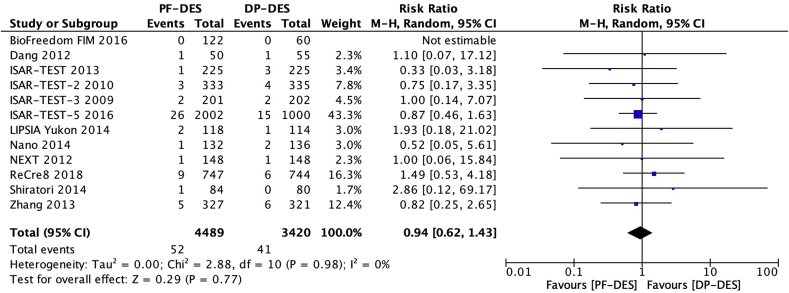
Risk of definite or probable stent thrombosis. DP-DES = durable polymer drug-eluting stents. M-H = Mantel-Haenszel. PF-DES = polymer-free drug-eluting stents.

Twelve trials [[16], [17], [18], [19], [20], [21], [22],[24], [25], [26], [27], [28]] reported definite or probable stent thrombosis (ST) in 7909 patients. There was no significant difference between patients receiving PF-DES and those receiving DP-DES for the risk of definite or probable ST (1.2% vs 1.2%; RR, 0.94; 95% CI, 0.62–1.43; *P* = 0.77; I^2^ = 0%; Fig. 2).

### 3.4 Myocardial infarction

**Fig. 3:**
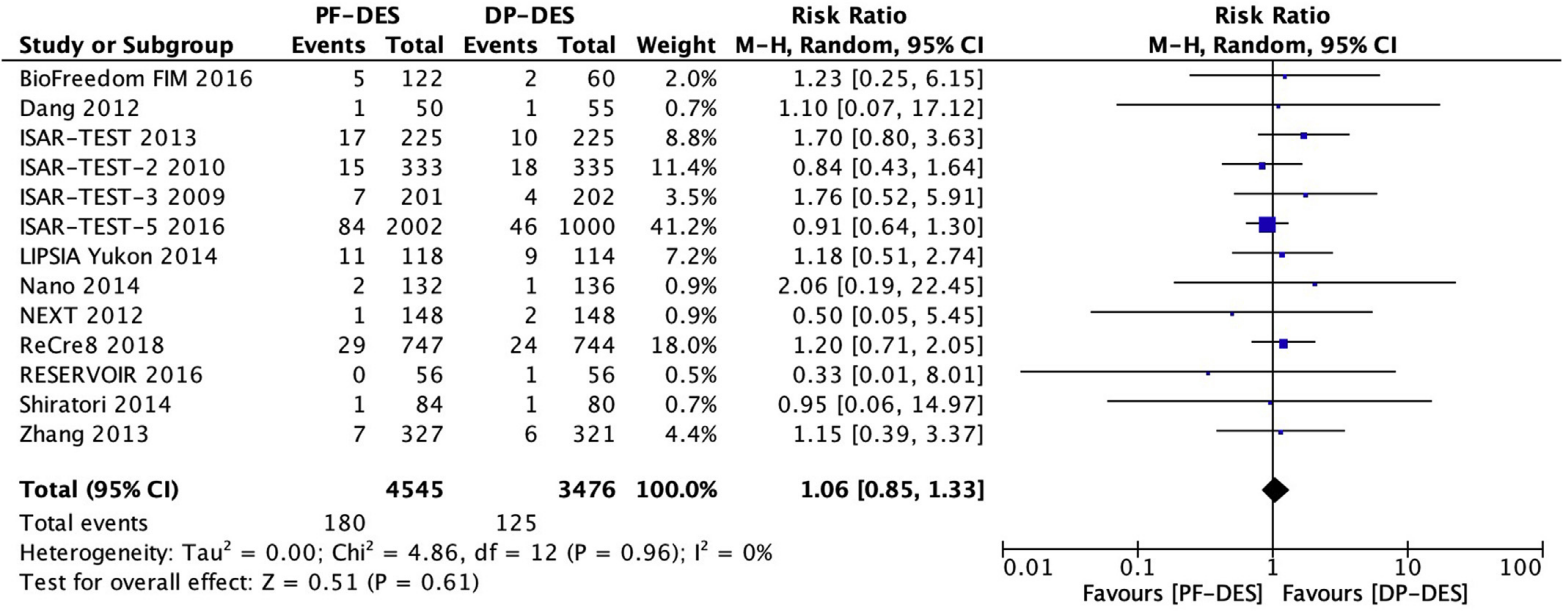
Risk of myocardial infarction. DP-DES = durable polymer drug-eluting stents. M-H = Mantel-Haenszel. PF-DES = polymer-free drug-eluting stents.

Thirteen trials [[16], [17], [18], [19], [20], [21], [22], [23], [24], [25], [26], [27], [28]] reported myocardial infarction (MI) in 8021 patients. There was no significant difference between patients receiving PF-DES and those receiving DP-DES for the risk of MI (4.0% vs 3.6%; RR, 1.06; 95% CI, 0.85–1.33; *P* = 0.61; I^2^ = 0%; Fig. 3).

### 3.5 Cardiac death

**Fig. 4:**
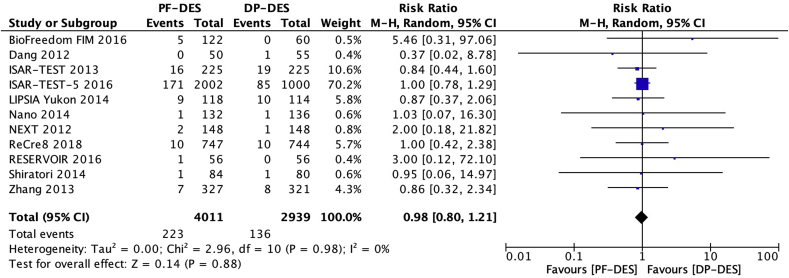
Risk of cardiac death. DP-DES = durable polymer drug-eluting stents. M-H = Mantel-Haenszel. PF-DES = polymer-free drug-eluting stents.

Eleven trials [[18], [19], [20], [21], [22], [23], [24], [25], [26], [27], [28]] reported cardiac death in 6950 patients. There was no significant difference between patients receiving PF-DES and those receiving DP-DES for the risk of cardiac death (5.6% vs 4.6%; RR, 0.98; 95% CI, 0.80–1.21; *P* = 0.88; I^2^ = 0%; Fig. 4).

### 3.6 All-cause death

**Fig. 5:**
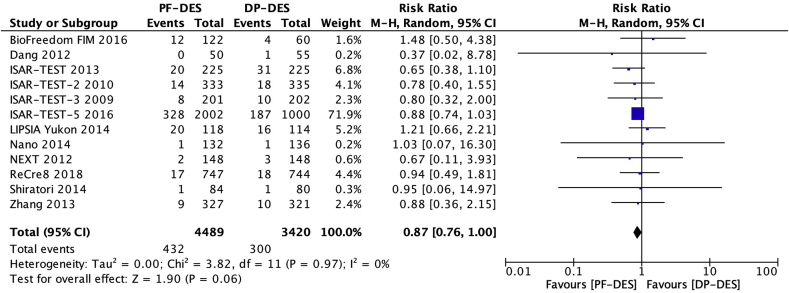
Risk of all-cause death. DP-DES = durable polymer drug-eluting stents. M-H = Mantel-Haenszel. PF-DES = polymer-free drug-eluting stents.

Twelve trials [[16], [17], [18], [19], [20], [21], [22],[24], [25], [26], [27], [28]] reported all-cause death in 7909 patients. There was no significant difference between patients receiving PF-DES and those receiving DP-DES for the risk of all-cause death (9.6% vs 8.8%; RR, 0.87; 95% CI, 0.76–1.00; *P* = 0.06; I^2^ = 0%; Fig. 5).

### 3.7 Target lesion revascularization

**Fig. 6:**
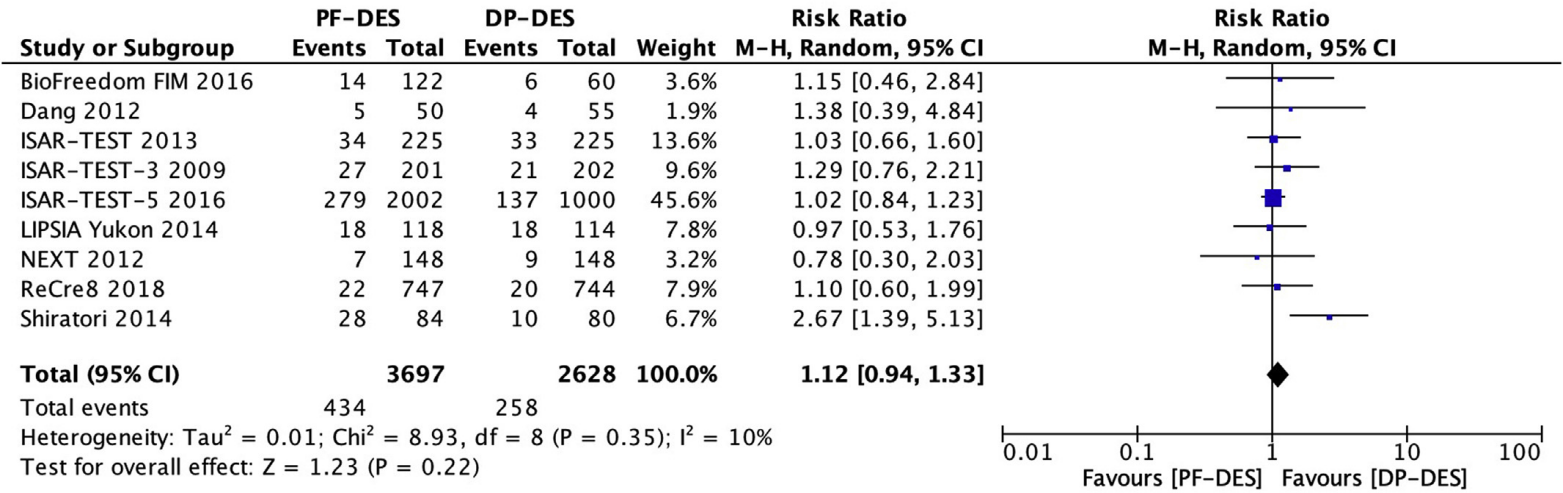
Risk of target lesion revascularization. DP-DES = durable polymer drug-eluting stents. M-H = Mantel-Haenszel. PF-DES = polymer-free drug-eluting stents.

Nine trials [[17], [18], [19], [20], [21], [22],[24], [25], [26]] reported target lesion revascularization (TLR) in 6325 patients. There was no significant difference between patients receiving PF-DES and those receiving DP-DES for the risk of TLR (11.7% vs 9.8%; RR, 1.12; 95% CI, 0.94–1.33; *P* = 0.22; I^2^ = 10%; Fig. 6).

### 3.8 Target vessel revascularization

**Fig. 7:**
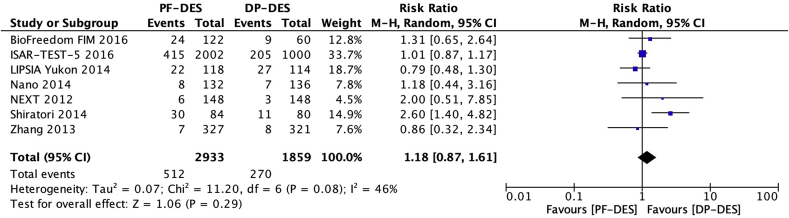
Risk of target vessel revascularization. DP-DES = durable polymer drug-eluting stents. M-H = Mantel-Haenszel. PF-DES = polymer-free drug-eluting stents.

Seven trials [18,19,21,22,25,26,28] reported target vessel revascularization (TVR) in 4792 patients. There was no significant difference between patients receiving PF-DES and those receiving DP-DES for the risk of TVR (17.5% vs 14.5%; RR, 1.18; 95% CI, 0.87–1.61; *P* = 0.29; I^2^ = 46%; Fig. 7).

### 3.9 Subgroup analyses

Table 3 outlines the safety and efficacy outcomes in different subgroups. At 1 year, 2 years, and 5 years of follow-up, there were no significant differences between patients with PF-DES and those with DP-DES for the risks of definite or probable ST, MI, cardiac death, all-cause death, TLR, and TVR (all *P* > 0.05). In addition, our landmark analysis beyond 1 year of follow-up showed no significant differences between patients with PF-DES and those with DP-DES for all outcomes (all *P* > 0.05). Our subgroup analysis based on PF-DES anti-proliferative drug (amphilimus, biolimus, paclitaxel, sirolimus, or sirolimus/probucol) showed no significant differences between patients with PF-DES and those with DP-DES for the risks of definite or probable ST, MI, cardiac death, all-cause death, and TVR (all *P* > 0.05). A similar result was observed in the risk of TLR for all PF-DES anti-proliferative drugs except for paclitaxel. Two trials [20,25] reported TLR in 134 patients receiving polymer-free paclitaxel-eluting stents (PF-PES) and 135 patients receiving DP-DES. Patients with PF-PES had a significantly increased risk of TLR than those with DP-DES (24.6% vs 10.4%; RR, 2.32; 95% CI, 1.30–4.14; *P* = 0.005; I^2^ = 0%). Our subgroup analyses based on DP-DES anti-proliferative drug (everolimus, paclitaxel, sirolimus, or zotarolimus), generation of DP-DES (first-generation or second-generation) and duration of DAPT (6 months or 12 months) showed no significant differences between patients with PF-DES and those with DP-DES for all outcomes (all *P* > 0.05).

**Table 3:** Safety and efficacy outcomes.

Analysis	Definite or Probable ST	MI	Cardiac Death	All-Cause Death	TLR	TVR
Outcomes at longest follow-up	0.94 (0.62–1.43)	1.06 (0.85–1.33)	0.98 (0.80–1.21)	0.87 (0.76–1.00)	1.12 (0.94–1.33)	1.18 (0.87–1.61)
Outcomes at 1 year	1.38 (0.56–3.43)	1.12 (0.68–1.85)	1.08 (0.50–2.32)	0.87 (0.48–1.60)	1.04 (0.65–1.67)	2.00 (0.51–7.85)
Outcomes at 2 years	0.85 (0.39–1.82)	1.06 (0.64–1.73)	0.88 (0.36–2.16)	0.82 (0.52–1.29)	1.81 (0.89–3.68)	1.52 (0.75–3.08)
Outcomes at 5 years	0.91 (0.50–1.69)	0.96 (0.70–1.32)	1.01 (0.79–1.28)	0.90 (0.77–1.06)	1.02 (0.85–1.21)	1.00 (0.87–1.15)
Landmark analysis beyond 1 year	0.34 (0.01–8.20)	0.44 (0.13–3.50)	0.46 (0.24–81.57)	0.85 (0.45–1.62)	1.09 (0.51–2.33)	1.07 (0.43–2.66)
Subgroup analysis						
PF-DES anti-proliferative drug						
Amphilimus	1.42 (0.54–3.73)	1.12 (0.67–1.87)	1.15 (0.52–2.54)	0.90 (0.49–1.67)	1.00 (0.60–1.65)	2.00 (0.51–7.85)
Biolimus	NR	1.23 (0.25–6.15)	5.46 (0.31–97.06)	1.48 (0.50–4.38)	1.15 (0.46–2.84)	1.31 (0.65–2.64)
Paclitaxel	0.97 (0.35–2.70)	1.12 (0.43–2.87)	0.81 (0.33–2.00)	0.84 (0.37–1.90)	*2.32 (1.30–4.14)	1.61 (0.55–4.71)
Sirolimus	0.67 (0.17–2.60)	1.52 (0.92–2.50)	0.86 (0.52–1.42)	0.84 (0.59–1.21)	1.09 (0.81–1.46)	0.85 (0.55–1.34)
Sirolimus/Probucol	0.85 (0.47–1.52)	0.90 (0.66–1.22)	1.00 (0.78–1.29)	0.87 (0.74–1.02)	1.02 (0.84–1.23)	1.01 (0.87–1.17)
DP-DES anti-proliferative drug						
Everolimus	NR	0.33 (0.01–8.01)	3.00 (0.12–72.10)	NR	NR	NR
Paclitaxel	0.77 (0.17–3.55)	1.43 (0.75–2.72)	0.97 (0.54–1.74)	0.75 (0.48–1.19)	1.29 (0.76–2.20)	1.93 (0.23–3.02)
Sirolimus	0.81 (0.38–1.72)	1.03 (0.62–1.71)	0.80 (0.31–2.07)	0.80 (0.51–1.27)	1.30 (0.80–2.14)	0.86 (0.32–2.34)
Zotarolimus	1.01 (0.59–1.72)	0.99 (0.74–1.33)	1.00 (0.79–1.28)	0.88 (0.75–1.03)	1.02 (0.85–1.23)	1.01 (0.87–1.17)
Generation of DP-DES						
First-generation	0.85 (0.44–1.64)	1.19 (0.83–1.69)	0.91 (0.59–1.39)	0.86 (0.65–1.14)	1.23 (0.93–1.64)	1.29 (0.83–2.02)
Second-generation	1.01 (0.59–1.72)	0.98 (0.73–1.32)	1.01 (0.80–1.28)	0.88 (0.75–1.03)	1.02 (0.85–1.23)	1.01 (0.87–1.17)
Duration of DAPT						
6 months	0.85 (0.48–1.53)	1.01 (0.75–1.38)	0.99 (0.79–1.25)	0.86 (0.74–1.00)	1.18 (0.87–1.60)	1.48 (0.87–2.51)
12 months	1.04 (0.57–1.91)	1.12 (0.80–1.56)	0.94 (0.57–1.56)	0.94 (0.69–1.30)	1.12 (0.81–1.57)	0.86 (0.57–1.29)

Values are risk ratio (95% confidence interval); MI = myocardial infarction; NR = not reported; PF-DES = polymer-free drug-eluting stents; TLR = target lesion revascularization; TVR = target vessel revascularization; ST = stent thrombosis; * = statistical significance (*P* < 0.05).

## 4 Discussion

The present meta-analysis of 13 RCTs compared the safety and efficacy profiles between PF-DES and DP-DES in a total of 8021 patients with coronary artery disease. Our findings demonstrated that the two stent platforms had similar rates of definite or probable ST, MI, cardiac death, all-cause death, TLR, and TVR. Overall, there were no identifiable safety and efficacy advantages of PF-DES over DP-DES. A meta-analysis [29] of 6178 patients found no significant differences between PF-DES and DP-DES for the risk of definite or probable ST at both short- (≤1 year) (odds ratio [OR], 0.95; 95% CI 0.54–1.67; *P* = 0.43) and long-term (>1 year) follow-up (OR, 0.75; 95% CI, 0.36–1.55; *P* = 0.53). Similar outcomes were observed for the risks of mortality, TLR, and TVR at both short- and long-term follow-up (all *P* > 0.05).

PF-DES were introduced with the aim to overcome the risks of late safety and efficacy outcomes associated with the preceding generations of devices. The polymer coating used in DP-DES has been shown to cause chronic inflammation and delayed vascular healing [30]. A meta-analysis [31] of 6575 patients reported that PF-DES had a significantly reduced risk of all-cause death compared with DP-DES (OR, 0.77; 95% CI, 0.61–0.98; *P* = 0.03). The present meta-analysis showed that the reduced risk of all-cause death associated with PF-DES was attenuated, and no longer reached statistical significance, with minimal heterogeneity between studies. This finding may be attributed to the inclusion of recent RCTs [23,24] comparing PF-DES with second-generation DP-DES. The new generation of DP-DES were found to have a significantly reduced risk of all-cause death compared with first-generation DP-DES (RR, 0.58; 95% CI, 0.37–0.90; *P* = 0.01) [32].

The polymer coating in DP-DES has multiple functions, such as stabilizing and binding the anti-proliferative drug to the stent platform and slowing down the rate of drug elution [33]. The ongoing challenge in the development of PF-DES has been maintaining these functions, while improving biocompatibility without the polymer coating. In general, PF-DES have a microporous metallic stent platform and an inorganic coating that can be loaded with an anti-proliferative drug [8]. These modifications in stent design and structure compared to preceding generations of devices will affect the elution profile of different anti-proliferative drugs [34]. Hence, we performed subgroup analyses based on PF-DES anti-proliferative drug (amphilimus, biolimus, paclitaxel, sirolimus, or sirolimus/probucol) and DP-DES anti-proliferative drug (everolimus, paclitaxel, sirolimus, or zotarolimus), which showed no significant differences between PF-DES and DP-DES for all outcomes except for TLR in PF-PES. Our subgroup analyses found that PF-PES had a significantly increased risk of TLR compared with DP-DES. This finding might not reflect a true difference due to the small sample size from two trials [20,25]. Nevertheless, a trial [35] of 1043 patients with PF-PES showed that approximately 40% of the anti-proliferative drug was lost from the stent surface during delivery of the device. This issue in drug release kinetics causes non-uniform local drug distribution, abnormal neointimal hyperplasia, and in-stent restenosis, which may contribute to the increased risk of TLR associated with PF-PES, as observed in our sensitivity analyses.

Second-generation DP-DES were shown to have improved clinical outcomes compared with first-generation DP-DES [6]. Our subgroup analysis based on generation of DP-DES found no significant differences for all outcomes. However, this finding should be interpreted with caution due to the small sample size. The comparison of safety and efficacy outcomes between PF-DES and second-generation DP-DES was derived from three trials [[22], [23], [24]].

The proposed rationale for developing PF-DES was to improve vascular healing in the stented segment, resulting in a shorter duration of DAPT following stent implantation [36]. However, our subgroup analysis based on duration of DAPT (6 months or 12 months) found no significant differences between PF-DES and DP-DES for all outcomes. The latest American College of Cardiology and American Heart Association (ACC/AHA) guidelines [7] have reduced the recommended duration of DAPT from 12 months to 6 months in patients receiving DES. There have been suggestions that short-term (1 month) DAPT may be safe following PF-DES implantation [37]. The LEADERS FREE trial [38] of 2466 patients found that PF-DES were superior to BMS with respect to primary safety endpoint (composite of definite or probable ST, MI, and cardiac death; hazards ratio [HR], 0.80; 95% CI, 0.64–0.99; *P* < 0.039) and primary efficacy endpoint (TLR; HR, 0.54; 95% CI, 0.41–0.72; *P* < 0.0001) when used with 1-month DAPT. The elimination of the polymer coating in PF-DES is thought to reduce the risk of very late ST compared with DP-DES. However, this theoretical benefit was not demonstrated at 1 year, 2 years, and 5 years of follow-up, as well as in our landmark analysis beyond 1 year of follow-up, which showed no significant difference between PF-DES and DP-DES for the risk of definite or probable ST.

In the present meta-analysis, electronic database searches were limited to RCTs to reduce the risk of bias. However, there were several limitations that deserve consideration. Firstly, as with any meta-analysis, our study should be interpreted in light of the limitations in design and quality of the original studies, which often compared stents with various anti-proliferative drugs, durations of DAPT, and lengths of follow-up. To address this source of bias, we performed subgroup analyses based on these variables. A network meta-analysis comparing different types of DES may be considered for future research when further trials become available. Secondly, the original studies did not report outcomes according to baseline characteristics, so we were unable to perform subgroup analyses to determine if these variables might influence the results. Thirdly, the exclusion of data from unpublished RCTs may have reduced the potential number of patients in each comparison arm. Fourthly, some reports of the original studies did not describe the randomization techniques, which made it difficult to assess the risk of bias. Finally, trials with greater length of follow-up in larger number of patients are necessary to evaluate the long-term safety and efficacy profiles of PF-DES compared with DP-DES. Further trials with shortened duration of DAPT are warranted to realize the theoretical benefits of PF-DES.

## 5 Conclusions

In summary, our findings demonstrated similar safety and efficacy profiles between PF-DES and DP-DES. The two stent platforms had equivalent safety and efficacy outcomes, including comparable rates of definite or probable ST.

## Funding sources

This research did not receive any specific grant from funding agencies in the public, commercial, or not-for-profit sectors.

## Provenance and peer review

Not commissioned externally peer reviewed.

## Ethical approval

This research does not contain any studies with human participants or animals.

## Sources of funding

None declared.

## Author contribution

James Wu: study design, data collection, data analysis, writing.

Joshua Way: study design, data collection, data analysis, writing.

Leonard Kritharides: study design, writing.

David Brieger: study design, writing.

## Conflicts of interest

None declared.

## Research registration number

reviewregistry619.

## Guarantor

James Wu.

David Brieger.
