# Supplementary material for: Polymer-free versus durable polymer drug-eluting stents in patients with coronary artery disease: A meta-analysis
Source: Ann Med Surg (Lond). 2018 Dec 11;38:13–21. doi: 10.1016/j.amsu.2018.12.003 (PMC6302254; doi:10.1016/j.amsu.2018.12.003)
Supplement: Multimedia component 1 [file mmc1.docx]

**Supplementary Table 1.** Assessment of risk of bias.

| **Study** | **Random Sequence Generation** | **Allocation Concealment** | **Blinding of Participants** | **Blinding of Outcome Assessment** | **Incomplete Outcome Data** | **Selective Reporting** | **Other Bias** |
| --- | --- | --- | --- | --- | --- | --- | --- |
| BioFreedom FIM 2016 (19) | Unclear | Unclear | Unclear | Low | Low | Low | Low |
| Dang 2012 (20) | Low | Low | Unclear | Unclear | Low | Unclear | Low |
| ISAR-TEST 2013 (21) | Low | Low | Unclear | Low | Low | Low | Low |
| ISAR-TEST-2 2010 (16) | Low | Low | Unclear | Low | Low | Low | Low |
| ISAR-TEST-3 2009 (17) | Low | Low | Low | Low | Low | Low | Low |
| ISAR-TEST-5 2016 (22) | Unclear | Low | Low | Low | Low | Low | Low |
| LIPSIA Yukon 2014 (26) | Low | High | Unclear | Low | Low | Low | Low |
| Nano 2014 (27) | Low | Unclear | Unclear | Low | Low | Low | Low |
| NEXT 2012 (18) | Low | Low | Unclear | Low | Low | Low | Low |
| ReCre8 2018 (24) | Low | Low | Unclear | Low | Low | Low | Low |
| RESERVOIR 2016 (23) | Low | Low | Low | Low | Low | Low | Low |
| Shiratori 2014 (25) | Low | Unclear | Low | Low | Low | Low | Low |
| Zhang 2013 (28) | Low | Low | Unclear | Unclear | Low | Unclear | Low |
